# Development and Psychometric Testing of the Mental Health Scale for Childrearing Fathers

**DOI:** 10.3390/healthcare9111587

**Published:** 2021-11-19

**Authors:** Aya Kondou, Mari Haku, Toshiyuki Yasui

**Affiliations:** 1 Department of Midwifery, Institute of Biomedical Sciences, Tokushima University Graduate School, Tokushima 770-8509, Japan; kondou.aya@tokushima-u.ac.jp; 2 Department of Reproductive and Menopausal Medicine, Institute of Biomedical Sciences, University Graduate School, Tokushima 770-8509, Japan; tosyasui@tokushima-u.ac.jp

**Keywords:** paternal depression, paternal childrearing, paternal mental health, anxiety, postpartum depression, scale development

## Abstract

The mental health of fathers influences the development of children and the functioning of families significantly. However, there is no useful scale for the mental health screening of childrearing fathers. This study developed a Mental Health Scale for Childrearing Fathers (MSCF) and determined its reliability and validity. Childrearing fathers are working fathers who co-parent with their spouses. This survey was conducted in two stages: a pilot study and a main survey. Data were obtained from 98 fathers raising preschoolers in the pilot study and 306 fathers in the main survey. The collected data were used to confirm the construct validity, criterion-related validity, convergent validity, and internal consistency reliability. The final MSCF consisted of 25 items comprising four factors: peaceful familial connection, healthy mind and body, satisfying paternal alliances, and leading a meaningful life as a parent. The internal consistency reliability estimated using Cronbach’s alpha coefficient for the total scale was 0.918. The validity of the MSCF was logically secured using a confirmatory factor analysis. The MSCF can be an effective tool for mental health screening among fathers in relation to the burden of childrearing during regular infant health checks.

## 1. Introduction

Anxiety and stress while childrearing tend to cause mental health issues, such as irritability, fatigue, and depressed mood. These can further lead to marital conflict, decreased affection between couples [1], child abuse [2,3], and mental health problems among children [4]. However, previous related studies show that anxiety and stress among childrearing mothers can be reduced by support from the children’s fathers [5].

Although Japan continues to promote gender equality and diversity, the country still upholds strong traditional gender roles, meaning, “men should work outside, whereas women should protect their families”. Therefore, in 2000, the government initiated a project to promote childrearing among fathers [6]. Owing to the recent promotion of childrearing among fathers in Japan, fathers are frequently observed accompanying mothers to regular infant health checks. According to a survey that targeted the time allocated for childrearing among men, those in the United States and Sweden devoted 71 min and 67 min, respectively, to daily childcare [7]. In Japan, fathers devote only 49 min to childcare daily [8]. Men work for an average of 7 h and 32 min, 5 h and 32 min and 5 h and 13 min per day in Japan, the United States, and Sweden, respectively [9]. Particularly, Japanese men work longer hours per day than men in other countries and attend to/care for their children briefly after work. Therefore, fathers often experience exhaustion and anxiety when raising their children [10].

Although postpartum depression-related problems mainly affect mothers within one year of childbirth, fathers also experience mental health issues. This is evidenced through a meta-analysis on paternal depression, which revealed a mean paternal depression rate of 10.4%, which is the highest rate of such depression three to six months postpartum [11]. Another meta-analysis showed that the rate of paternal depression reached 7.8%, 13.0%, 10.0%, and 11.8% at 0–3, 3–6, 6–9, and 9–12 months postpartum, respectively [12]. The prevalence of perinatal depression among Japanese men was 8.5%, 13.2%, and 8.2% before delivery, between 3 and 6 months postpartum, and 6 to 12 months postpartum, respectively [13]. In a longitudinal study on men across 23 years, although the men who became fathers exhibited decreased symptoms of depression before childbirth, this rate increased by 68% on average during 0–5 years of having a child [14]. Moreover, since the COVID-19 outbreak in December 2019, the increased risk of domestic violence [15,16] and child abuse [17] continues to affect family harmony significantly. In a survey on the effects of pregnancy, childbirth, and childrearing, previous scholars indicated that women suffered increased symptoms of anxiety and depression during the pandemic [18,19,20,21]. Similarly, fathers experienced a significant increase in stress levels during the pandemic, particularly in relation to childrearing, compared with the stress experienced by mothers, and fathers equally require support during this period [22].

Screening and understanding the mental health of childrearing fathers are significant tasks of healthcare providers owing to the significant effect of these tasks on the development of children and functioning of families [23,24]. However, thus far, no suitable scale to screen the mental health of childrearing fathers has been developed.

Therefore, this study aimed to develop a Mental Health Scale for Childrearing Fathers (MSCF) and to determine its reliability and validity.

## 2. Materials and Methods

**Figure 1:**
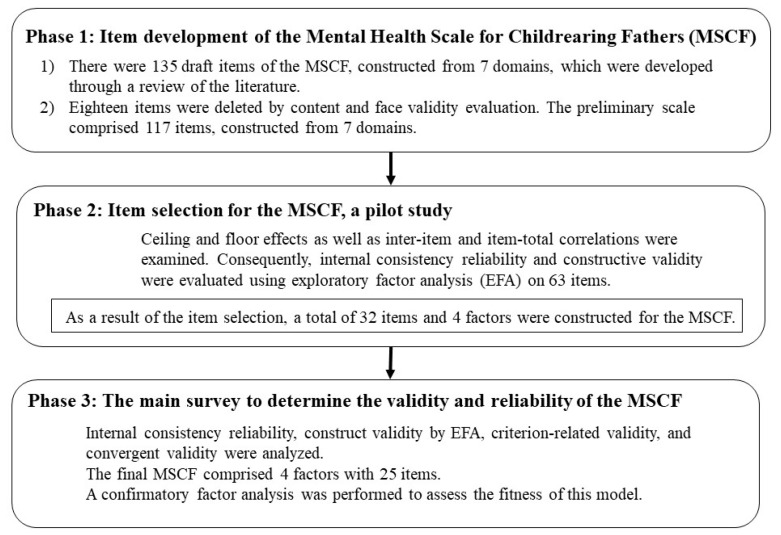
Flow chart for the development of the Mental Health Scale for Childrearing Fathers.

The development of the MSCF comprises three steps: the item development of the MSCF based on a review of the literature, item selection, and conducting the main survey to determine the validity and reliability of the MSCF (Figure 1).

### 2.1. Phase 1: Item Development of the MSCF

The MSCF was developed based on a review of the literature. Subsequently, a scale comprising the following domains was drafted:

1. Daily happiness: Subjective wellbeing encompasses two aspects: recognition and emotion. The components of subjective wellbeing are pleasant emotions, unpleasant emotions, and self-satisfaction in life [25]. A total of 14 items were used to evaluate satisfaction in terms of the living environment and feelings, such as pleasure or overall fulfillment in life.
2. Self-esteem: Sakurai [26] asserted that a psychologically unhealthy person has low self-esteem. Moreover, Rosenberg [27] reported that self-esteem comes from within. The present study used 11 items to evaluate the acceptance of, and value for, oneself.
3. Family stability: Family members influence one another. Paternal depression has a specific and persistent harmful effect on the early behavioral and emotional development of children [23]. Paternal depression is negatively associated with marital relationships [24]. The relationship with the wife and interfamily reliability comprised 22 items.
4. Feelings toward childrearing: Childrearing evokes positive and negative feelings. Nelson et al. [28] asserted that parents exhibit relatively higher levels of happiness, positive emotions, and meaning in life than nonparents, which indicates positive feelings. In contrast, anxiety, and the burden of childrearing indicate negative feelings. The present study used 35 items to evaluate this dimension.
5. Social support: Low levels of social support were significantly associated with depression among fathers [29]. However, high levels of social support seemingly buffer the effect of depression on negative life events and diseases [30,31]. Perceived social support for caring and kindness was evaluated using seven items.
6. Work satisfaction: One of the risk factors of paternal depression was unstable employment, such as temporary employment and unemployment [32]. Job satisfaction, fulfillment, accomplishment, and human relations associated with work were evaluated using 15 items.
7. Physical and mental health: According to the ICD-10, depression is characterized by three key symptoms, namely, persistent sadness or negative mood, loss of interest or pleasure, and fatigue or low energy. Other common symptoms include disturbed sleep, poor concentration or indecisiveness, low levels of self-confidence, poor or decreased appetite, suicidal thoughts or acts, agitation or slowed movements, and guilt or self-blame [33]. Therefore, mental and physical health are related; in other words, depression affects physical health, which, consequently, drives depression. Anxiety, sadness, fatigue, and disturbed sleep constituted 31 items.

These seven domains are considered to be related to each other. Based on these seven domains, the MSCF comprises 135 items.

### 2.2. Examining Content Validity and Face Validity for the MSCF Draft

Three expert midwifery educators and two practicing midwives with more than 10 years of experience in mother and child health-related matters read and reviewed all items of the questionnaire to examine instrument content validity. All items that were approved, rejected, and modified were based on a consensus. Twelve fathers involved in childrearing answered all items and identified awkward or unclear items by examining face validity. Eighteen items were eliminated. The preliminary scale for the pilot study comprised 117 out of 135 items for the final version.

### 2.3. Phase 2: Item Selection of the MSCF and the Pilot Study

A pilot study was conducted between January and May 2019. A total of 150 fathers from one hospital were selected for this study, and 98 (65.3%) fathers completed the questionnaire, while 52 (34.7%) did not. The average age of the total valid sample was 35.8 ± 4.9 years (27–51 years).

They were all married, in good health, above 20 years of age, and raising a preschooler. The fathers received a package containing the explanation of the study, a self-administered questionnaire, and an envelope for returning replies. When the fathers were unavailable, their wives took the package home to their husbands. Once the questionnaires were completed, the fathers mailed them back to the research team.

The pilot study included the developed preliminary scale of the MSCF and data on age, occupation, mean working hours, family type, and the number and age of the participants’ children. The items for the preliminary scale of the MSCF were rated on a four-point Likert scale. Higher scores indicated better mental health.

### 2.4. Phase 3: Item Selection of the MSCF and the Main Survey

The main survey was conducted between January and July 2020. We recruited 568 participants from one hospital, one healthcare center, and seven nursery schools. The total number of valid samples was 306, with a response rate of 53.9%. A total of 262 participants did not complete the questionnaire (46.1%).

The same survey process and content in the pilot study were used for the main survey. To examine criterion-related validity and convergent validity, five additional self-administered questionnaires (see below) were issued to 130 out of the total 568 respondents. The total number of valid samples was 92, with a response rate of 70.8%. Thirty-eight (29.2%) participants did not complete the questionnaire.

### 2.5. Scales for Determining Criterion-Related Validity and Convergent Validity

#### 2.5.1. The Quality of Marriage Index (QMI)

The QMI was developed by Norton [34] and translated into Japanese by Moroi [35]. The reliability of the Japanese version of this scale has been examined previously (α = 0.927). It comprises six items on the quality of marriage and uses a four-point Likert scale, with higher scores indicating better quality of marriage. The Cronbach’s α coefficient for this study was 0.884.

#### 2.5.2. Self-Esteem

The Rosenberg Self-Esteem Scale [27] was translated into Japanese by Sakurai [26]. The reliability of the Japanese version of this scale has been examined previously (α = 0.84). It is a self-evaluating scale that assesses self-acceptance. It comprises 10 items and uses a four-point Likert scale, with higher scores indicating higher levels of self-esteem. The Cronbach’s α coefficient for this study was 0.858.

#### 2.5.3. The Center for Epidemiologic Studies-Depression (CES-D) Scale

The CES-D scale was developed by Radloff [36] and translated into Japanese by Shima et al. [37]. The reliability of the Japanese version of this scale has been examined previously (rt = 0.794, Spearman–Brown’s formula). It evaluates both physical and mental symptoms of depression. It comprises 20 items and uses a four-point Likert scale, with higher scores indicating higher levels of depression. The Cronbach’s α coefficient for this study was 0.782.

#### 2.5.4. The 12-Item General Health Questionnaire (GHQ12-J)

The GHQ12-J was developed by Goldberg [38] and translated into Japanese by Nakagawa et al. [39]. The reliability of the Japanese version of this scale has been examined previously (α = 0.89). It comprises 12 items on health conditions and uses a four-point Likert scale, with higher scores indicating worse mental health. The Cronbach’s α coefficient for this study was 0.865.

#### 2.5.5. The Subjective Wellbeing Inventory (SUBI-J)

The SUBI-J evaluates wellbeing based on the World Health Organization’s (WHO) definition of health [40]. It was translated into Japanese by Ono et al. [41]. The scale distinguishes between positive (19 items) and negative (21 items) affects. The reliability of the Japanese version of this scale in determining positive affect (α = 0.86–0.89) and negative affect (α = 0.84–0.86) has been examined previously. It uses a three-point Likert scale, with higher scores for positive affect indicating higher levels of wellbeing. Higher scores for negative affect represent lower levels of illbeing. The Cronbach’s α coefficient for positive and negative affect in this study was 0.896 and 0.808, respectively.

### 2.6. Procedure for Data Analysis

In Phase 2 (the pilot study), the demographic data of the participants were evaluated using descriptive statistics. The ceiling and floor effects as well as inter-item and item–total correlations were analyzed. Ceiling and floor effects were ascertained by eliminating items with a mean ± standard deviation of ≥4.0 or ≤1.0. The inter-item correlation of all items was analyzed and set to ≥0.7. Items that displayed similarities were excluded. An item with an item–total correlation of less than 0.4 was considered an inappropriate question, and consequently eliminated. In the first factor analysis, the number of factors from the eigenvalue, scree plot, and cumulative contribution rate were determined. Before the factor analysis, the Kaiser–Meyer–Olkin (KMO) and Bartlett’s test of sphericity confirmed the validity of the samples.

An exploratory factor analysis (EFA) was conducted using the principal factor method and promax rotation. Factor loadings equal to or greater than 0.4 were considered appropriate. Cronbach’s α coefficients were used to confirm the reliability of the scale.

In Phase 3 (the main survey), item analysis and EFA were repeated with data from a larger sample. Moreover, criterion-related validity and convergent validity were calculated using the Spearman’s rank correlation coefficients. A confirmatory factor analysis (CFA) was performed to assess the fitness of the model. Fit indices, including the goodness of fit index (GFI), adjusted goodness of fit index (AGFI), comparative fit index (CFI), root mean square error of approximation (RMSEA), and Akaike’s information criterion (AIC), were used to assess the fitness of the model. Bagozzi and Yi reported that a GFI ≥ 0.90 and an AGFI ≥ 0.90 are recommended [42]. The value of CFI could range between 0 and 1, and the values closer to 1 indicate data fitness [43]. Browne and Cudeck reported that RMSEA with values less than 0.08 indicate an acceptable fit to the data [44]. AIC adopts smaller values when the models considered good are compared with multiple models [45]. Cronbach’s α coefficients confirmed the reliability of the scale. Statistical analyses were conducted using SPSS Statistics version 25 and Amos version 26 (IBM Corp, Tokyo, Japan).

### 2.7. Ethical Considerations

The participants were provided a written explanation regarding the purpose of the study. Participation consent was based on their responses to the questionnaires, their mailing them back to the research team, and the receipt of their answers. Participation was voluntary. There were no penalties or disadvantages for refusal to participate. The participants understood that confidentiality would be maintained and that data would not be used for any purpose other than that of the study. The results were statistically presented without individual identification. The study was approved by the Ethics Committee of Tokushima University Hospital (Approval No. 3252).

## 3. Results

### 3.1. Participant Characteristics

In the main survey, the average age was 37.5 ± 5.9 years. Full-time employees accounted for 83.7% of the respondents. The mean working hours of the participants were 9.3 ± 1.5 h. In total, 37.6% of the participants had one child, and 48.0% had two children.

The MSCF focused on working fathers co-parenting with a spouse rather staying at home fathers (Table 1).

### 3.2. Phase 2

#### Item Selection for the MSCF (Pilot Study)

Ceiling effect was observed for seven items that reflected social desirability, whereas floor effect was noted for six items on mental symptoms. A total of 13 out of 117 items were omitted. Regarding inter-item correlation, the correlations of 104 items were calculated, and the contents of the items were examined. Consequently, 22 items that exhibited similarities were eliminated. Regarding the item–total correlation, items with low correlation tend to reduce the reliability of the scale, such that 19 items below 0.4 were eliminated. EFA was further conducted on 63 items (KMO = 0.696; Bartlett’s test of sphericity was significant at *p* < 0.001). Only items with factor loading ≥0.4 were included in the questionnaire, and 31 items were eliminated. As a result of EFA, the total number of items was 32. The Cronbach’s α coefficient for the scale was 0.918; for the subscales, the Cronbach’s α coefficients ranged from 0.785 to 0.924, demonstrating good internal consistency.

### 3.3. Phase 3

#### Construct Validity and Reliability (Main Survey)

Correlations between the 32 items were analyzed. When inter-item correlations were 0.7 or more and the item contents were similar, the average score and standard deviation for each question item were examined. A criterion was further set to eliminate the item with the larger standard deviation. If the standard deviations were identical, the question items were examined, and the question items that the respondents found difficult to understand were eliminated. The items with the highest correlation coefficients were as follows. The correlation coefficient between Q10 and Q11, Q11 and Q12, Q16 and Q17, Q20 and Q21, and Q23 and Q24 was 0.74, 0.80, 0.71, 0.79, and 0.79, respectively. Four items (Q.12, Q17, Q21, and Q23) were eliminated. Q11 was retained as it was judged to be an item that cross-examined the relationship with the wife, which is considered to affect the fathers’ mental health significantly. The item–total correlations were further examined. A low correlation coefficient lowers the reliability of the scale. One item (Q18) was eliminated. Consequently, 27 items remained (Table 2).

The result of KMO reached 0.912, and Bartlett’s test of sphericity was significant (*p* < 0.001). In the first analysis, four factors with an eigenvalue of over 1.00 and a cumulative contribution rate of over 50.0% were identified. After EFA, two items (Q4 and Q7) were excluded. Consequently, the total number of items was 25. The study identified four factors: peaceful familial connection, healthy mind and body, satisfying paternal alliances, and leading a meaningful life as a parent.

In the main survey, the Cronbach’s α coefficient for the total scale was 0.918 and ranged from 0.792 to 0.897 for the subscales (Table 3).

**Figure 2:**
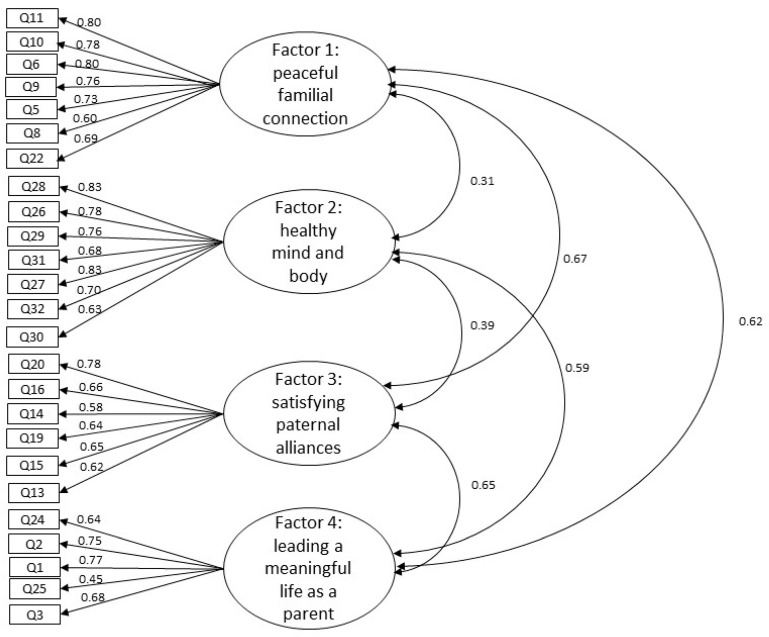
Confirmatory factor analysis of the MSCF based on model A in Table 4. Note: MSCF = Mental Health Scale for Childrearing Fathers. The path coefficient values calculated from the latent variables to the observable variables were statistically significant (*p* < 0.001). Correlations between latent variables were also statistically significant (*p* < 0.001).

The CFA determined the appropriateness of the fit of the hypothetical model obtained in the EFA. Twenty-five items were subjected to the CFA. Model A (GFI = 0.828, AGFI = 0.792, CFI = 0.873, RMSEA = 0.078), which contained items for evaluating the mental health of childrearing fathers and was the most applicable (Table 4), was adopted. The path coefficient values calculated from the latent variables to the observable variables were statistically significant (0.45–0.83, *p* < 0.001). Correlations between latent variables were also statistically significant (0.31–0.67, *p* < 0.001) (Figure 2).

### 3.4. Criterion-Related Validity and Convergent Validity

The total score for the MSCF was significantly and positively correlated with the score of positive affect under the SUBI-J (*r* = 0.72, *p* < 0.01), and significantly and negatively correlated with the GHQ12-J score (*r* = −0.79, *p* < 0.01). Moreover, the results exhibited strongly significant positive correlations between the total scores of the first factor (peaceful familial connection) and the QMI score (*r* = 0.71, *p* < 0.01), the fourth factor (leading a meaningful life as a parent), and the score for positive affect under the SUBI-J (*r* = 0.74, *p* < 0.01). By contrast, the second factor (healthy mind and body) and CES-D score exhibited a negative correlation (*r* = −0.71, *p* < 0.01) (Table 5).

## 4. Discussion

The final scale of the MSCF consisted of 25 items comprising four factors: peaceful familial connection, healthy mind and body, satisfying paternal alliances, and leading a meaningful life as a parent.

Although there are other scales for evaluating mental health [36,38], there are no questionnaires on the mental health needs of childrearing fathers. To our knowledge, the MSCF is a new scale centered on paternal mental health. It evaluates a childrearing father’s mental health while considering all aspects of his life, including employment status, emotional state, and marital status. Therefore, the MSCF could provide a much more profound and accurate depiction of a father’s mental state related to the burden of childrearing. The EPDS is a mental health scale used by postnatal women, which includes questions on mental health [46]. Among the second factor of the MSCF, there are items similar to the EPDS. However, the third and fourth factors of the MSCF include items specific to childrearing fathers and are considered an original scale.

The scale’s reliability was evaluated for internal consistency using Cronbach’s α coefficient. The Cronbach’s α coefficient for the total scale was 0.918, and over 0.79 for the four subscales. The Cronbach’s α coefficient of 0.7 or higher is acceptable [47]. Therefore, the internal consistency of the MSCF is well above the alpha criterion.

In phases 2 and 3, the KMO values reached 0.696 and 0.912, respectively, and Bartlett’s test of sphericity was significant (*p* < 0.001) for both phases. High KMO values (close to 1.0) indicate that factor analysis is useful for our data. Additionally, small values of Bartlett’s test of sphericity (less than 0.05 level of significance) indicate that factor analysis is useful for our data.

The first factor was referred to as “a peaceful familial connection”, which comprises seven items (Table 3). This factor comprised the item that indicates the positive feelings associated with couple relations and family functioning that changed after having children. Scholars report that paternal depression is associated with lower marital relationships, which influences family functioning [24,48]. This factor can evaluate the mental health of fathers in terms of marital and family relationships.

The second factor was referred to as “the healthy mind and body”, which comprises seven items (Table 3). All items for this factor reflected negative feelings. The regulation of negative emotional expressions is associated with poor mental health [49,50]. Alternatively, the suppression of emotional expression is positively correlated with depression [51]. The study considered that the second factor could be used to evaluate the feelings of fathers, which contribute to the suppression of the depressive state through the expression of feelings.

The third factor was referred to as “satisfying paternal alliances”, which comprises six items (Table 3). This factor comprised items that indicate happiness and satisfaction while living with the child and taking up the role of a parent. Being a father is fun, enjoyable, and rewarding [52]. Scholars indicate that fathers should discipline and raise their children responsibly [53]. The item “I think everything is progressing well, especially in terms of being a father” was intended to evaluate self-esteem. A previous related study reported that a psychologically unhealthy person exhibits low self-esteem [26]. This factor can screen the aspects of wellbeing and adaptability, which can lead to the examination of the support required to encourage positive perspectives toward childrearing.

The fourth factor was referred to as “leading a meaningful life as a parent”, which comprised five items (Table 3). All items reflected positive feelings. Fathers face the dilemma of balancing their work responsibilities with spending time with their children [54]. This factor can screen the perspective of fathers about life with children as they attempt to maintain a balance between work and family.

The evidence of criterion-related validity and convergent validity is supported by the following notions. The MSCF displayed a strong positive correlation with QMI for evaluating marital relationships, and the SUBI-J assesses the state of total physical, mental, and social wellbeing. Moreover, the MSCF exhibited a strong negative correlation with CES-D, which is used to identify high-risk groups for depression symptoms, and GHQ12-J, which evaluates physical and mental health. Therefore, the MSCF includes items that exclusively evaluate not only physical and mental conditions but also family relationships (the first factor) and life as a father (the fourth factor).

The MSCF was positively and strongly correlated with positive affect in the SUBI-J, owing to its use of several items for positive feelings, with high scores indicating better general wellbeing. By contrast, the MSCF was negatively and strongly correlated with CES-D, with high scores indicating depression. Therefore, the study confirmed that the MSCF includes items to screen for anxiety and depression among fathers, in relation to the burden of childrearing.

The CFA was conducted on the factors mentioned above and covered 25 items. The model fit indicated that some of the values did not meet the acceptability criteria. However, the values of the model fit were very similar. Therefore, the scale’s validity is logically secured.

However, the MSCF is limited in that it does not apply to fathers without an occupation as it contains two items related to work. This study supports healthy fathers; therefore, healthcare providers should examine more methods of investigation to account for various psychosomatic situations and explore policies that provide help in future.

This study’s results have the following implications. The MSCF may be used during regular infant health checks. This may provide parents the opportunity for individual consultation with public health nurses about their anxieties and worries regarding childrearing, as well as the growth and development of their children. In individual consultations, public health nurses should also pay attention to the mental health of parents. Recently, in Japan, fathers have been observed to frequently accompany mothers to regular infant health checks, owing to the promotion of childrearing among fathers. Therefore, this study believes that public health nurses in charge of regular infant health checks can use the MSCF at the time of individual consultation to screen the mental state of fathers in relation to the burden of childrearing. The MSCF is relatively straightforward as it comprises only 25 items and can be completed in 10 min. Therefore, it can be made available to fathers during regular infant health checks. Furthermore, a comprehensive assessment of the mental health of fathers can be achieved by combining the MSCF with an interview.

## 5. Conclusions

The MSCF evaluates a father’s mental state related to the burden of childrearing while considering all aspects of his life, including employment status, emotional state, and marital status. The MSCF was developed by analyzing the literature, item pool design, expert modification, a pilot study, scale modification, and a main survey using EFA and CFA. The final scale consisted of 25 items comprising four factors: peaceful familial connection, healthy mind and body, satisfying paternal alliances, and leading a meaningful life as a parent. The reliability estimated using Cronbach’s α coefficient for the total scale was 0.918. The validity of the MSCF was logically secured using a confirmatory factor analysis. Therefore, the MSCF can be an effective tool for screening the mental status of fathers in relation to the burden of childrearing during regular infant health checks.

## Figures and Tables

**Table 1:** Participant characteristics.

Characteristics	Phase 2 (N = 98)	Phase 3 (N = 306)
	*n* (%)	
**Age (years)** (mean ± SD)	35.8 ± 4.9	37.5 ± 5.9
Occupation		
Full-time employee	85 (86.7)	256 (83.7)
Loan employee	0 (0.0)	1 (0.3)
Contracted employee	4 (4.1)	10 (3.3)
Part-time employee	1 (1.0)	1 (0.3)
Temporary employee	0 (0.0)	2 (0.7)
Other (self-employed, farmer etc.)	8 (8.2)	36 (11.8)
Mean working hours (mean ± SD)	9.5 ± 1.9	9.3 ± 1.5
Family type		
Nuclear family	78 (79.6)	276 (90.2)
Extended family	20 (20.4)	30 (9.8)
Number of children		
One child	28 (28.6)	115 (37.6)
Two children	53 (54.1)	147 (48.0)
Three children or more	17 (17.3)	44 (14.4)
Age of the eldest child (years) (mean ± SD)	4.7 ± 3.4	4.9 ± 3.6
Age of the youngest child (years) (mean ± SD)	2.1 ± 2.0	2.5 ± 1.9

**Table 2:** Item analysis of the MSCF (N = 306).

Item Number	Items	Mean ± SD	Inter-Item Correlation	Item-Total Correlation
Q1 (Domain 1)	I feel good about my life.	3.32 ± 0.67	−0.39–0.62	0.66 **
Q2 (Domain 1)	I am hopeful about my future.	2.93 ± 0.77	−0.39–0.62	0.60 **
Q3 (Domain 1)	I lead a meaningful life.	3.02 ± 0.82	−0.38–0.52	0.64 **
Q4 (Domain 2)	I am confident in accomplishing the task.	3.07 ± 0.80	−0.35–0.42	0.52 **
Q5 (Domain 3)	I feel comfortable when my family is together.	3.57 ± 0.59	−0.23–0.61	0.54 **
Q6 (Domain 3)	I consider my family united.	3.33 ± 0.70	−0.24–0.67	0.63 **
Q7 (Domain 3)	I am considerate toward my family.	3.32 ± 0.62	−0.27–0.52	0.55 **
Q8 (Domain 3)	My family life is peaceful.	3.31 ± 0.69	−0.29–0.56	0.55 **
Q9 (Domain 3)	My family feels good with each other.	3.15 ± 0.73	−0.24–0.67	0.61 **
Q10 (Domain 3)	My family supports me when necessary.	3.40 ± 0.73	−0.22–0.74	0.55 **
Q11 (Domain 3)	I feel happy about my relationship with my wife.	3.40 ± 0.75	−0.21–0.80	0.56 **
Q12 ^a^ (Domain 3)	I am content with my wife.	3.31 ± 0.83	−0.23–0.80	-
Q13 (Domain 3)	I do my best as a husband.	2.91 ± 0.73	−0.27–0.64	0.53 **
Q14 (Domain 4)	I feel happy when I am with my child.	3.77 ± 0.45	−0.22–0.53	0.44 **
Q15 (Domain 4)	I am content with the relationship I have with my child.	3.55 ± 0.57	−0.23–0.53	0.53 **
Q16 (Domain 4)	I consider childrearing a worthwhile task.	3.29 ± 0.72	−0.23–0.71	0.50 **
Q17 ^a^ (Domain 4)	I feel good about childrearing.	3.24 ± 0.72	−0.25–0.71	-
Q18 ^b^ (Domain 4)	I am at ease regarding my child’s growth.	3.78 ± 0.49	−0.13–0.46	0.34 **
Q19 (Domain 4)	I think everything is progressing well, especially in terms of being a father.	2.85 ± 0.79	−0.28–0.48	0.55 **
Q20 (Domain 4)	I feel my reason for living is being a father.	3.32 ± 0.73	−0.28–0.79	0.64 **
Q21 ^a^ (Domain 4)	I am happy to be a father.	3.32 ± 0.77	−0.25–0.79	-
Q22 (Domain 5)	I have a valuable person, who can share in my happiness and sadness.	3.29 ± 0.79	−0.29–0.55	0.62 **
Q23 ^a^ (Domain 6)	I feel a sense of achievement in my work.	2.82 ± 0.89	−0.41–0.79	-
Q24 (Domain 6)	I am content with my work.	2.67 ± 0.89	−0.49–0.79	0.57 **
Q25 (Domain 6)	I think accomplishing my task leads to my personal growth.	2.99 ± 0.85	−0.29–0.56	0.45 **
Q26 (Domain 7)	I don’t feel at ease (R).	2.05 ± 0.88	−0.35–0.67	−0.54 **
Q27 (Domain 7)	I am dispirited (R).	1.98 ± 0.90	−0.49–0.69	−0.67 **
Q28 (Domain 7)	I am anxious(R).	2.25 ± 0.97	−0.38–0.69	−0.60 **
Q29 (Domain 7)	I lose my concentration (R).	1.83 ± 0.77	−0.33–0.64	−0.59 **
Q30 (Domain 7)	I feel nervous (R).	1.85 ± 0.82	−0.33–0.58	−0.57 **
Q31 (Domain 7)	I am rushed (R).	2.06 ± 0.94	−0.35–0.58	−0.59 **
Q32 (Domain 7)	I am reluctant to have thoughts (R).	1.94 ± 0.89	−0.35–0.60	−0.58 **

Note: Spearman’s rank correlation coefficient test. ** *p* < 0.01. ^a^ Items were eliminated after inter-item correlation analysis. ^b^ The item was eliminated after item-total correlation analysis. MSCF = Mental Health Scale for Childrearing Fathers. R = reverse scoring. The numbers in parentheses show the initial domains each question belonged to.

**Table 3:** Exploratory factor analysis of the MSCF (N = 306).

Factor and Cronbach’s α	Item Number	Items	Factor Loading			
			1	2	3	4
First factor: Peaceful familial connection **α = 0.890**	Q11	I feel happy about my relationship with my wife.	**0.952**	0.048	−0.163	−0.028
	Q10	My family supports me when necessary.	**0.834**	0.032	0.009	−0.081
	Q6	I consider my family united.	**0.766**	0.018	−0.017	0.081
	Q9	My family feels good with each other.	**0.689**	0.023	0.068	0.035
	Q5	I feel comfortable when my family is together.	**0.648**	−0.017	0.213	−0.110
	Q8	My family life is peaceful.	**0.562**	−0.140	0.042	−0.072
	Q22	I have a Valuable person, who can share in my happiness and sadness.	**0.541**	−0.027	0.163	0.067
Second factor: Healthy mind and body **α = 0.897**	Q28	I am anxious (R).	0.059	**0.843**	0.043	−0.038
	Q26	I don’t feel at ease (R).	0.045	**0.839**	0.061	0.035
	Q29	I lose my concentration (R).	−0.061	**0.762**	0.015	0.026
	Q31	I am rushed (R).	−0.100	**0.730**	−0.040	0.127
	Q27	I am dispirited (R).	0.057	**0.711**	0.032	−0.225
	Q32	I am reluctant to have thoughts (R).	0.051	**0.686**	−0.028	−0.035
	Q30	I feel nervous (R).	−0.081	**0.628**	−0.144	0.117
Third factor: Satisfying paternal alliances **α = 0.815**	Q20	I feel my reason for living is being a father.	0.058	−0.007	**0.719**	0.050
	Q16	I consider childrearing a worthwhile task.	0.057	0.073	**0.649**	0.009
	Q14	I feel happy when I am with my child.	0.122	0.049	**0.616**	−0.111
	Q19	I think everything is progressing well, especially in terms of being a father.	−0.105	−0.131	**0.598**	0.083
	Q15	I am content with the relationship I have with my child.	0.056	−0.003	**0.594**	0.054
	Q13	I do my best as a husband.	−0.027	−0.062	**0.544**	0.099
Fourth factor: Leading a meaningful lifeas a parent **α=0.792**	Q24	I am content with my work.	−0.167	−0.058	−0.010	**0.822**
	Q2	I am hopeful about my future.	0.128	0.004	−0.013	**0.679**
	Q1	I feel good about my life.	0.329	0.028	0.013	**0.535**
	Q25	I think accomplishing my task leads to my personal growth.	−0.164	0.127	0.291	**0.516**
	Q3	I lead a meaningful life.	0.324	−0.124	−0.072	**0.423**
**Total Cronbach’s α coefficient was 0.918.**						

Note: Bold represents the significant data. An exploratory factor analysis was conducted using the principal factor method and promax rotation. The KMO was 0.912, and Bartlett’s test of sphericity was significant (*p* < 0.001). MSCF = Mental Health Scale for Childrearing Fathers. R = reverse scoring.

**Table 4:** The model fitness index of the MSCF for the underlying model A and alternative models B, C, and D (N = 306).

Model	*χ^2^*	*df*	GFI	AGFI	CFI	RMSEA	AIC
A	764.691 ***	269	0.828	0.792	0.873	0.078	876.691
B	788.303 ***	271	0.822	0.786	0.867	0.079	896.303
C	789.604 ***	270	0.827	0.791	0.867	0.079	899.604
D	802.207 ***	270	0.823	0.787	0.863	0.080	912.027

Note: Structural equation modeling was used for the analysis; AIC = Akaike information criterion; AGFI = adjusted goodness of fit index; CFI = comparative fit index; *df* = degrees of freedom; GFI = goodness-of-fit index; MSCF = Mental Health Scale for Childrearing Fathers; RMSEA = root mean square error of approximation; *χ^2^* = chi-square; *** *p* < 0.001. Model A was 25 items of the MSCF by the exploratory factor analysis. Model B was a second-order model of the MSCF with a mental health factor underpinning factors 1, 2, 3, and 4. Model C had no covariance between factors 1 and 2 in Model A. Model D had no covariance between factors 2 and 3 in Model A.

**Table 5:** Correlations among the MSCF and five scales (N = 92).

	Total MSCF Items	First Factor: Peaceful Familial Connection	Second Factor: Healthy Mind and Body	Third Factor: Satisfying Paternal Alliances	Fourth Factor: Leading a Meaningful Life as a Parent
QMI	0.57 **	0.71 **	0.31 **	0.23	0.52 **
Self-esteem	0.64 **	0.34 **	0.58 **	0.42 **	0.58 **
CES-D	−0.65 **	−0.31 **	−0.71 **	−0.32 **	−0.58 **
GHQ12-J	−0.79 **	−0.64 **	−0.67 **	−0.39 **	−0.65 **
SUBI-J					
Positive affect	0.72 **	0.61 **	0.49 **	0.43 **	0.74 **
Negative affect	0.55 **	0.31 **	0.65 **	0.29 **	0.36 **

Note: Spearman’s rank correlation coefficient test. ** *p* < 0.01. CES-D = Center for Epidemiologic Studies-Depression; GHQ12-J = The 12-Item General Health Questionnaire; MSCF = Mental Health Scale for Childrearing Fathers; QMI = The Quality of Marriage Index; SUBI-J = The Subjective Wellbeing Inventory.

## Data Availability

The data that support the findings of this study are available from the corresponding author upon reasonable request.
